# Non-strangulated Spigelian Hernia: A Case Report

**DOI:** 10.7759/cureus.27699

**Published:** 2022-08-05

**Authors:** Rangin Haji Rahman, Anila Punjwani, Janna Notario-Ringwald, Srishti Taneja, Sarwan Fahim, Rahul Varghese, Frederick Tiesenga

**Affiliations:** 1 General Surgery, Saint James School of Medicine, Park Ridge, USA; 2 Neurology, Avalon University School of Medicine, Youngstown, USA; 3 General Surgery, West Suburban Medical Center, Chicago, USA

**Keywords:** abdominal hernia, ipom plus, ipom, mesh, laparoscopic surgery, abdominal surgery, spigelian, hernia

## Abstract

Spigelian hernia is a rare type of ventral hernia with an incidence of 0.1-2%. We report a case of a non-strangulated left lower quadrant spigelian hernia and its management. A 74-year-old female presented with progressively worsening left flank pain along with dysuria and frequency related to pyelonephritis. Incidentally, CT of the abdomen and pelvis demonstrated a left spigelian hernia containing intermediate size small bowel without strangulation. Thereafter, she began developing increasing abdominal pain in that area. The hernia was repaired on the same day as admission via laparoscopic intraperitoneal onlay mesh-plus repair. Spigelian hernia possesses an elusive clinical presentation. Though rare, it must be considered in the differential diagnosis of abdominal hernia due to its high risk for acute complications.

## Introduction

Spigelian hernia is a rare variation of ventral hernia, representing 0.1-2% of all abdominal hernia cases [1]. A spigelian hernia is the herniation or protrusion of preperitoneal fat, peritoneal sac, or organ(s) through a defect in the spigelian aponeurosis, which is the aponeurotic layer between the rectus abdominis muscle medially, and the semilunar line laterally. The spigelian aponeurosis/fascia, formed by the fusion of the internal oblique and transversus abdominis aponeurosis, is an inherently weak area of the abdominal wall. This increases the likelihood of developing a hernia through this region under conditions of increased intraabdominal pressure [2]. These predisposing factors include obesity, chronic obstructive pulmonary disease (COPD), abdominal trauma, and abdominal surgery [1]. While most cases of spigelian hernia are acquired, cases of congenital spigelian hernia have been documented in the literature. This is thought to be due to weak areas in the aponeurosis of the abdominal muscles formed during embryological development.

Clinically, this type of hernia is able to evade detection and palpation on a physical exam due to its location deep in the external oblique aponeurosis [3]. As such, patients may present in the urgent setting secondary to acute complications such as incarceration, strangulation, or bowel obstruction. In terms of management, this kind of hernia is not amenable to conservative treatment due to the high likelihood of complications [3]. Operative repair remains the standard of care in urgent and non-urgent settings. In this paper, we report a case of a left lower quadrant spigelian hernia that was successfully treated using a laparoscopic approach.

## Case presentation

A 74-year-old female with a medical history of obesity, hypertension, diabetes mellitus, pulmonary sarcoidosis, transient ischemic attack, and prior abdominal surgery (bladder surgery, hysterectomy, and tubal ligation) presented to the emergency department with progressively worsening left flank pain over the course of several days along with dysuria and frequency. She exhibited constitutional symptoms of fever, chills, and sweats, as well as complained of nausea and abdominal pain. On physical examination, mild tenderness was elicited on palpation of the left flank. IV ceftriaxone was administered in the acute setting.

**Figure 1:**
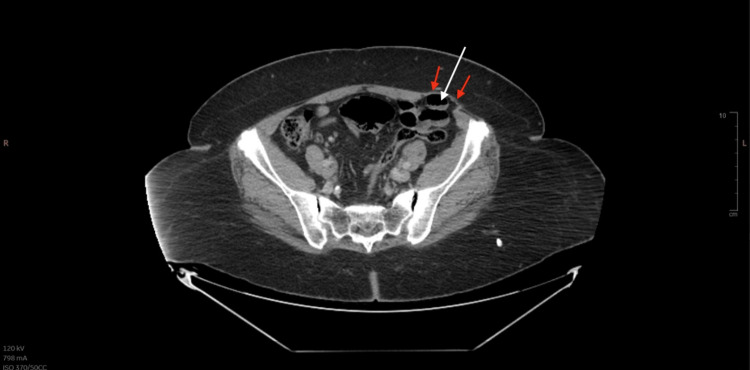
CT of the abdomen and pelvis post-contrast displaying a left spigelian hernia with small bowel content (white arrow). Also seen is the intact aponeurosis of the external abdominal oblique (red arrows).

Laboratory studies demonstrated the following data: total white cell count 23,400 WBC/μL (N: 4500-11,000 WBC/μL), hemoglobin 11.9 g/dL, platelets 163,000 platelets/μL, sodium 137 mmol/L, potassium 3.4 mmol/L, and chloride 100 mmol/L. Computed tomography (CT) of the abdomen and pelvis with IV contrast incidentally revealed a left spigelian hernia containing an intermediate size small bowel (Figure 1). Over the course of her stay, she began developing increasing abdominal pain in the left lower quadrant where the hernia was localized, without evidence of abdominal swelling or lump. Also found on CT were pyelonephritis and bilateral nephrolithiasis, accounting for the patient’s initial chief complaint of left flank pain and the presence of constitutional symptoms. A recommendation was made for laparoscopic intraperitoneal onlay mesh (IPOM)-plus repair. The procedure was explained to the patient, and informed witnessed consent was obtained. 

The operation was performed under general and local anesthesia, and the patient received appropriate IV antibiotics within one hour of skin incision. Intraoperatively, the spring-loaded needle was placed in the left subcostal region at Palmer’s point, and the abdomen was insufflated to 15 mmHg. A 5 mm trocar was then placed in an Optiview (Ethicon, Raritan, New Jersey) fashion in the right upper quadrant, followed by two additional 5 mm trocars in the right hemiabdomen. Attention was placed to the left lower quadrant. There was a 3.5 cm defect just lateral to the inferior epigastric vessels, corresponding with the spigelian hernia visualized on CT. The underlying small bowel was viable without any evidence of perforation or gangrene. A decision was made for repair. A trocar site closure device with absorbable suture was used to place two figure-of-eight sutures to primarily close the defect, followed by the placement of a 13.5 cm mesh, which was then fixed to the abdominal wall with metallic spiral tacks. The hernia sac was dissected out and removed and sent as a specimen prior to closure. The operative field was hemostatic at the end of the case. Pneumoperitoneum was desufflated, followed by anesthetization with a local anesthetic. There were no intraoperative complications. Pathology confirmed that the specimen consisted of fibromembranous adipose tissue with mesothelium, consistent with a hernia sac. During the postoperative period, the patient experienced episodes of emesis on the second postoperative day and was made nil per os until transitioned to a liquid diet on the fourth postoperative day. A full liquid diet was started on the seventh postoperative day, and a solid diet was initiated on the eighth postoperative day. Pain medication was administered as needed as well as the continuation of IV vancomycin and ceftriaxone. Her WBC count decreased from the day of admission to the second postoperative day from 23,400 WBC/μL to 18,700 WBC/μL. This was an inadvertent effect of the vancomycin, which was discontinued and replaced with a course of ceftriaxone alone for 14 days. By the fifth postoperative day, her WBC count stabilized to 8000 WBC/μL, indicating that her active pyelonephritis was in a state of resolution. With a resolution of infection at 6900 WBC/μL and adequate healing of her surgical incision sites, she was discharged on the ninth postoperative day. Postoperatively, the patient was readmitted for reasons related to her genitourinary issues, with no hernia recurrences identified as of the time of writing.

## Discussion

Spigelian hernia is a rare type of hernia comprising 0.1-2% of all abdominal wall defects [1]. Although its incidence in men and women is nearly equal, it occurs more frequently in women and typically between the ages of 40-70 [1,4,5]. Moreover, it tends to localize on the left side [1,6], as was seen in this case report. Risk factors include obesity, COPD, abdominal surgery, abdominal trauma, and other causes of increased intraabdominal pressure [1]. In our report, it was known that the patient was morbidly obese as well as had a prior history of abdominal surgery, specifically bladder surgery, tubal ligation, and a hysterectomy.

From a clinical standpoint, the diagnosis is difficult to make as the symptoms are non-specific in the early stages, ranging from vague abdominal pain to a visible or palpable lump and even features of incarceration with or without features of strangulation [1,7,8]. This ambiguous and sometimes asymptomatic presentation is due to the masking of the defect by thick subcutaneous fat and the tough aponeurosis of the external oblique (Figure 1) [7]. This poses a diagnostic challenge as clinical signs typically emerge in the later stages as the herniation increases in size, subsequently increasing the risk of strangulation and associated complications [1]. Specifically, fibrous bands of spigelian fascia are capable of forming a “rigid neck”, which increases the risk of strangulation, and this risk is reported to be between 2-14% [7,9]. The risk of incarceration is reported to lie between 24-27% [7,10,11]. Therefore, imaging via CT or ultrasound is critical for early detection and prompt surgical intervention. CT is recommended to determine the location of the defect, size, and content of the hernia sac, which guides the surgical management [1]. In this patient’s case, she, too, exhibited an asymptomatic presentation that was likely complicated by her superimposed renal issues, making it difficult to discern the origin of the pain. It wasn’t until further along in her hospital admission that she started developing increasing abdominal pain in the left lower quadrant. Therefore, had her pyelonephritis not resulted in her hospital admission, the hernia likely would have been missed until acute complications had developed.

Surgery remains the mainstay of treatment for spigelian hernia, and repair can be performed through an open or laparoscopic approach, depending on the patient’s characteristics, type of hernia, and experience of the surgeon [12,13]. In the last 20 years, the laparoscopic approach has become the method of choice - not only for repair but as a definitive diagnostic tool in patients with equivocal findings on ultrasound and CT [8]. The laparoscopic approach also provides the added benefit of reducing the length of hospitalization, the likelihood of postoperative infection, and morbidity [8]. Laparoscopic techniques include IPOM, totally extraperitoneal patch (TEP) approach, transabdominal preperitoneal (TAPP) approach, and laparoscopic suturing techniques. Although there are no clear guidelines favoring one technique over the other, the most popular approach is IPOM due to its ease of learning and safety advantages [8]. According to recent European Hernia Society (EHS) guidelines, mesh repair is recommended regardless of whether an open or laparoscopic approach is selected, as it offers a low rate of recurrence [14,15]. A variation of the IPOM technique is the IPOM-plus, the surgical method chosen for our patient. It employs the same principles of IPOM with the addition of defect closure with suture, thus offering a lower rate of recurrence as compared to IPOM [16]. 

## Conclusions

Spigelian hernia lacks specific and consistent physical findings, thus making a delayed diagnosis a frequent occurrence. As such, a high index of clinical suspicion is required due to the potential for life-threatening complications. Our patient presented asymptomatically and was hospitalized for genitourinary infection at an opportune point in time, allowing for prompt repair of the hernia. CT imaging proved useful in establishing the diagnosis, and when imaging is inconclusive, diagnostic laparoscopy may provide almost one-hundred percent accuracy. According to the literature, operative repair remains the mainstay of treatment. In this case presentation, the optimal form of management was IPOM-plus repair with laparoscopy. Despite the rarity of spigelian hernia, it must be considered in the differential diagnosis of abdominal hernia due to its high risk for acute complications.
